# Complex chromosomal rearrangements by single catastrophic pathogenesis in NUT midline carcinoma

**DOI:** 10.1093/annonc/mdw686

**Published:** 2017-02-14

**Authors:** J.-K. Lee, S. Louzada, Y. An, S. Y. Kim, S. Kim, J. Youk, S. Park, S. H. Koo, B. Keam, Y. K. Jeon, J.-L. Ku, F. Yang, T. M. Kim, Y. S. Ju

**Affiliations:** 1 Korea Advanced Institute of Science and Technology, Graduate School of Medical Science and Engineering, Daejeon, Korea; 2 Molecular Cytogenetics Core Facility, Wellcome Trust Sanger Institute, Wellcome Genome Campus, Hinxton, Cambridge, UK; 3 Biomedical Science and Engineering Interdisciplinary Program, Korea Advanced Institute of Science and Technology, Daejeon; 4 Department of Laboratory Medicine, Chungnam National University School of Medicine, Daejeon; 5 Seoul National University Cancer Research Institute, Seoul; 6 Departments of Internal Medicine; 7 Pathology, Seoul National University Hospital, Seoul, Korea

**Keywords:** NUT midline carcinoma, chromoplexy, complex genomic rearrangement, mutational signature, bromodomain and extra terminal

## Abstract

**Background:**

Nuclear protein in testis (NUT) midline carcinoma (NMC) is a rare aggressive malignancy often occurring in the tissues of midline anatomical structures. Except for the pathognomonic *BRD3/4–NUT* rearrangement, the comprehensive landscape of genomic alterations in NMCs has been unexplored.

**Patients and methods:**

We investigated three NMC cases, including two newly diagnosed NMC patients in Seoul National University Hospital, and a previously reported cell line (Ty-82). Whole-genome and transcriptome sequencing were carried out for these cases, and findings were validated by multiplex fluorescence *in situ* hybridization and using individual fluorescence probes.

**Results:**

Here, we present the first integrative analysis of whole-genome sequencing, transcriptome sequencing and cytogenetic characterization of NUT midline carcinomas. By whole-genome sequencing, we identified a remarkably similar pattern of highly complex genomic rearrangements (previously denominated as chromoplexy) involving the *BRD3/4–NUT* oncogenic rearrangements in two newly diagnosed NMC cases. Transcriptome sequencing revealed that these complex rearrangements were transcribed as very simple *BRD3/4–NUT* fusion transcripts. In Ty-82 cells, we also identified a complex genomic rearrangement involving the *BRD4–NUT* rearrangement underlying the simple t(15;19) karyotype. Careful inspections of rearrangement breakpoints indicated that these rearrangements were likely attributable to single catastrophic events. Although the NMC genomes had >3000 somatic point mutations, canonical oncogenes or tumor suppressor genes were rarely affected, indicating that they were largely passenger events. Mutational signature analysis showed predominant molecular clock-like signatures in all three cases (accounting for 54%−75% of all base substitutions), suggesting that NMCs may arise from actively proliferating normal cells.

**Conclusion:**

Taken together, our findings suggest that a single catastrophic event in proliferating normal cells could be sufficient for neoplastic transformation into NMCs.

## Introduction

Nuclear protein in testis (NUT) midline carcinoma (NMC) is a rare lethal malignancy, which was first described in the literature in 1991 [1, 2]. NMC shows heterogeneous clinicopathologic characteristics [3]; it occurs without gender predilection in a wide range of ages at diagnosis from newborns to the 70s with a peak incidence in the late teenage years [3]. It usually presents with poorly differentiated carcinoma with a variable degree of squamous differentiation and tends to occur in the midline anatomical structures, most frequently in the upper aerodigestive tract and anterior mediastinum; however, cases originating from the bladder, salivary glands, and iliac bone have also been described [3]. NMCs are frequently diagnosed with metastatic disease and refractory to chemotherapy with extremely poor patient survival [4]. Because of the heterogeneous presentation overlapping with other malignancies and the aggressive clinical course, NMC cases could have been frequently misclassified as other malignancies [3]; thus, the true incidence is unknown, and it has only recently emerged as an independent diagnostic category defined by the characteristic genomic rearrangements [5].

The genetic hallmarks of NMCs are the rearrangement of the *NUT* gene with a set of partner genes. Interchromosomal translocations between chromosomes 15q and 19p are frequently observed [1, 2], which generates a fusion oncogene *Bromodomain containing 4* (*BRD4*)−*NUT* [6, 7]. Briefly, the BRD4*–*NUT fusion oncoprotein results in a global disruption of gene expression programs through extensive chromatin remodeling [8], overexpression of *MYC* [9] and sequestration of the acetylated (activated) form of p53 [10]. Other partners of the *NUT* gene rearrangement, i.e. *BRD3* [7] or *NSD3* [11], are functionally related with BRD4, indicating that the recruitment of NUT to the chromatin through the bromodomain and extra terminal (BET) family proteins is necessary for the pathogenesis of NMCs. In accordance with their functional importance, the use of small molecular inhibitors of BET family proteins suggested a new therapeutic avenue for NMCs [12].

Except for the pathognomonic gene rearrangements, however, the comprehensive mutational landscape of NMC has been unexplored. Here we present the first whole-genome and transcriptome sequences of NMC from two newly diagnosed NMC cases and a previously reported NMC cell line [13]. In-depth analysis of the somatic mutational profile provides insight into the mechanisms of *BRD3/4*−*NUT* rearrangements and the characteristics of the original cells.

## Materials and methods

### Establishment of patient-derived cell lines

The protocol of this study was reviewed and approved by the Institutional Review Board of Seoul National University Hospital (#H-1512-083-728). This study was conducted in the observation of the Helsinki Declaration of the World Medical Association. Pleural effusions obtained after written informed consent were centrifuged, washed, and resuspended in growth media and then cultured as previously described [14]. Through this process, SNU-2972-1 and SNU-3178S cells were established from the NMC1 and NMC2 patients, respectively.

### High-throughput sequencing and analysis of somatic mutations

Genomic DNA and mRNA were extracted from the early batch SNU-2972-1, SNU-3178S, their matched peripheral blood samples and from Ty-82 cells. Detailed procedures of high-throughput sequencing and somatic mutation calling were described in a separate document (supplementary text, available at *Annals of Oncology* online).

### Cytogenetic characterization and fluorescence *in situ* hybridization

Conventional cytogenetic examination using Giemsa banding was done for the SNU-2972-1 and SNU-3178S cells. More than 20 metaphase cells were analyzed, and their karyotypes were assessed according to the International System for Human Cytogenetic Nomenclature 2013.

M-FISH and bacterial artificial chromosome (BAC)-FISH were done as previously described [15]. A human 24-color chromosomal painting probe set was used for the M-FISH. For analysis, 10 randomly selected metaphase cells were karyotyped based on the M-FISH and DAPI-banding patterns. For detailed analyses of particular rearrangement segments, human BAC clones were selected based on their genomic positions in the reference genome. Detailed information on the BAC clones used in our experiments is summarized in supplementary Table S1, available at *Annals of Oncology* online.

### Analysis of mutational signatures

Decomposition of mutational signatures from consensus substitution sets of individual cases was done with deconstructSigs package [16] and further confirmed with an in-house algorithm (based on 30 mutational signatures; http://cancer.sanger.ac.uk/cosmic/signatures).

## Results

### Clinical history of patients and establishment of NMC cell lines

We investigated two newly diagnosed patients with NMC. In both cases, the definitive diagnosis was made by confirming the high expression of NUT protein with immunohistochemical staining of the biopsy tissue (anti-NUT antibody C52B1; Cell Signaling Technology). Patient 1 was a 34-year-old male with a 10 pack-year history of smoking and was diagnosed with sinonasal and intrathoracic NMC with left pleural effusion. His tumor was resistant to two different combination chemotherapy regimens: three cycles of paclitaxel and carboplatin, and one cycle of cyclophosphamide, doxorubicin and vincristine. The treatment-refractory pleural effusion, which was drained a few days before death was later established as a NMC cell line, SNU-2972-1. Patient 2 was a 33-year-old never-smoking female who was diagnosed with NMC in the lung with multiple bone metastases. The SNU-3178S cell line was established from her pleural effusion at the initial presentation. Her tumor was initially stabilized by the combination chemotherapy of paclitaxel and carboplatin but eventually progressed after five cycles of treatment. The overall survival durations from the diagnosis of the two patients were 4 and 8 months, respectively.

Giemsa band karyotyping revealed that these two NMC cell lines do not have the canonical t(15;19) karyotype (supplementary Figure S1, available at *Annals of Oncology* online). SNU-2972-1 had a near-diploid karyotype with a translocation between chromosomes 15 and 21, and an insertion of chromosomal 15 segments into chromosome 19. The SNU-3178S cells had a complex karyotype of t(9;15) with hyperdiploidy of chromosomes 1, 5, 8, 12 and X. These microscopic chromosomal rearrangements could be further characterized at nucleotide resolution with whole-genome and transcriptome sequencing. To minimize the *in vitro* culture-induced mutations, we sequenced the early batches of SNU-2972-1 (denoted as NMC1) and SNU-3178S (NMC2) together with their matched peripheral blood samples as controls.

### Complex rearrangements involving *BRD*−*NUT* oncogenes

**Figure 1.**
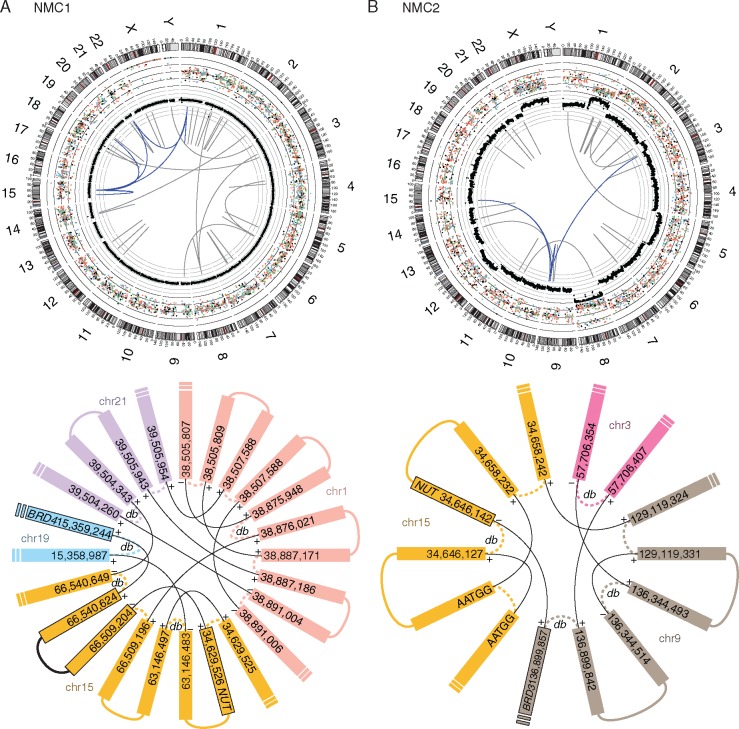
Chromoplexy generating the *BRD3/4*−*NUT* rearrangements. In the upper panels, genomic alterations of two cases (A; NMC1, B; NMC2) are described in circos plots. From the outside in, chromosomal ideograms and their genomic positions in megabases, base substitutions with their variant allelic fractions, smoothened copy numbers, and structural variations are plotted. Genomic rearrangements involved in the chromoplexy are highlighted in blue colors. For base substitutions, we use different colors for indicating different mutational spectra (C > A, blue; C > G, black; C > T, red; T > A, grey; T > C, green; T > G, pink). In the lower panels, the pattern of rearrangement and breakpoints of chromoplexy chains are described. Solid lines represent rearrangements, and dashed lines indicate DNA double strand breaks. >10-bp sized deletions overlying the double strand breaks are indicated as deletion bridges (db). Plus signs indicate rearrangement with reference strand, while minus signs indicate rearrangement with non-reference strand.

**Figure 2.**
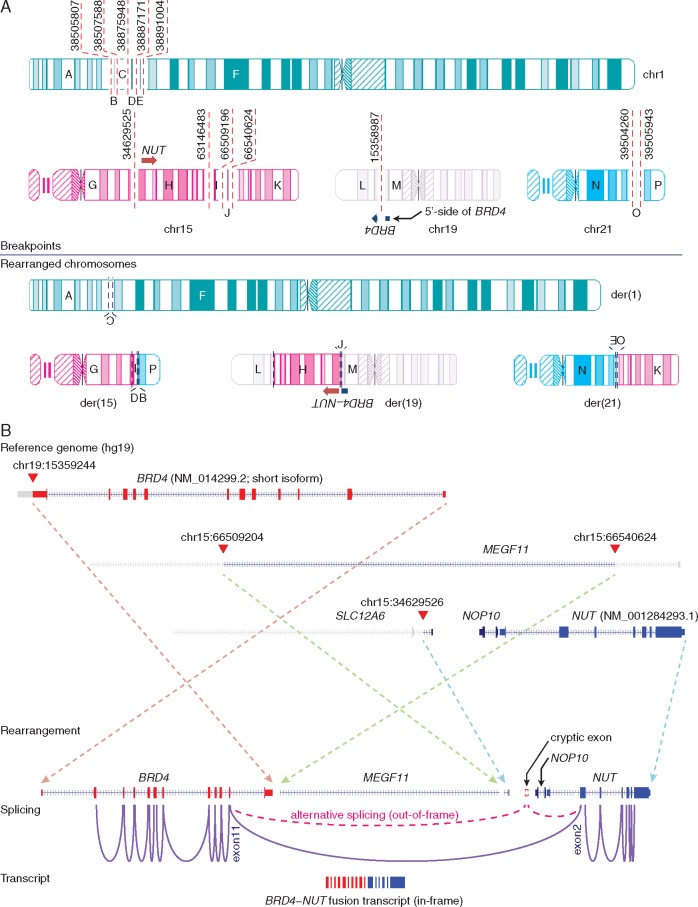
Pattern of chromosomal rearrangement involving the *BRD4*−*NUT* rearrangement and its transcription in NMC1. (A) Genomic breakpoints and their rearranged chromosomes in NMC1 are described. (B) Genomic rearrangement (arrows) in the vicinity of NUT gene and its pattern of transcription (purple lines) are represented.

**Figure 3.**
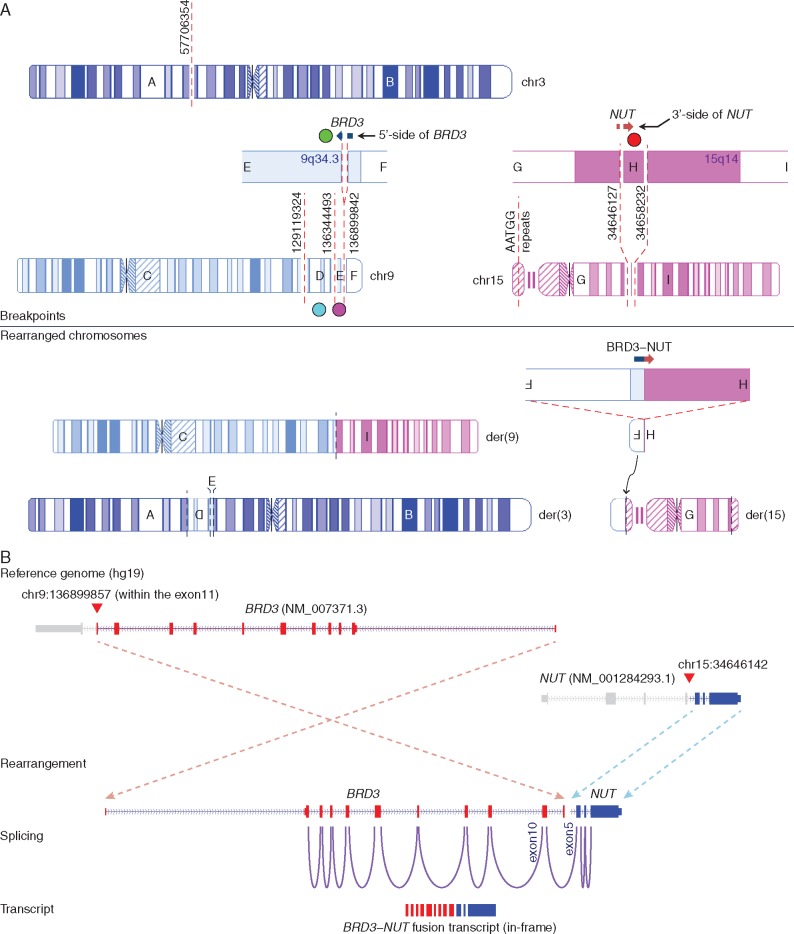
Pattern of chromosomal rearrangement involving the *BRD3*−*NUT* rearrangement and its transcription in NMC2. (A) Genomic breakpoints and the three derivative chromosomes in NMC2 are described. (B) *BRD3*−*NUT* rearrangement and its transcriptional pattern are shown.

**Figure 4.**
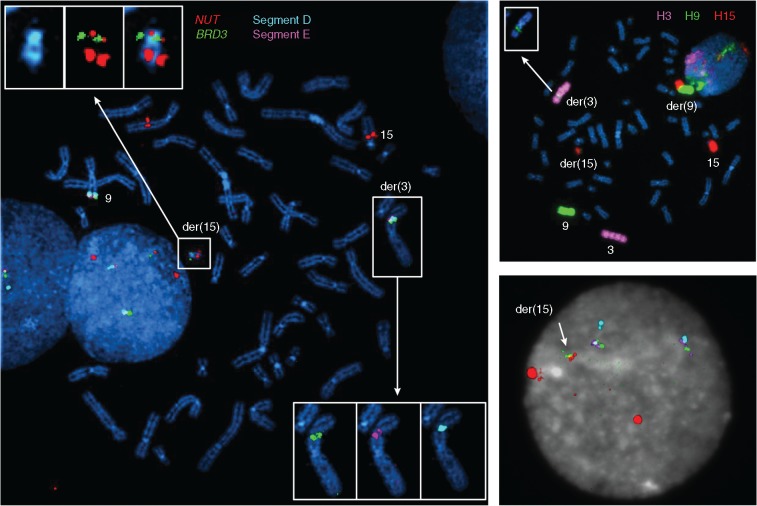
Identification of *BRD3*−*NUT* rearrangement located in the chromosome 15 by BAC-FISH in NMC2. To visualize the *BRD3*−*NUT* rearrangement of which the genomic location is unclear in whole-genome sequencing, we chose BAC clones corresponding to the genomic positions of segment D, E, *BRD3*, and *NUT* for individual probe FISH. BAC-FISH visualized the *BRD3*−*NUT* rearrangement located in the short arm of acrocentric chromosome 15 (left). All three derivative chromosomes are shown in individual paint probe FISH (right).

We first investigated large structural variations (SVs) including genomic rearrangements and DNA copy number alterations. With *en suite* bioinformatics analyses and visual inspections, we detected 49 and 33 highly confident cancer-specific SVs from NMC1 and NMC2, respectively (supplementary Table S2, available at *Annals of Oncology* online). The allele-specific copy number analysis of NMC1 and NMC2 showed highly concordant copy number profiles compared with their karyotypes (supplementary Table S3, available at *Annals of Oncology* online). Interestingly, the rearrangements in the vicinity of the *NUT* gene were highly complex in both tumors consisting of many inter- and intra-chromosomal breakpoints with no evidence of local DNA copy number alterations. A careful manual reconstruction of these rearranged segments revealed a remarkably similar “closed chain” pattern of complex genomic rearrangements across multiple chromosomes (Figure 1), previously described as chromoplexy [17]. Inspection of breakpoint pairs which were likely generated from single DNA double strand breakages revealed frequent >10-bp deletions as described previously [17]; these deletions may reflect the DNA resection during the repair process. In NMC1, 24 genomic breakpoints, clustered in several hotspots in chromosomes 1, 15, 19 and 21, were rejoined in a balanced but disordered manner to form a closed chain of 12 rearrangements. The complex event finally rearranged *BRD4* to the upstream of *NUT* with an intervening 31-kb-sized insertion segment from chromosome 15 (Figures 1A and 2A). These findings were validated by multiplex fluorescence *in situ* hybridization (M-FISH; supplementary Figure S2, available at *Annals of Oncology* online) and FISH using individual paint probes (supplementary Figure S3, available at *Annals of Oncology* online). The mRNA sequencing revealed the expression of the rearranged genome: exon 11 of *BRD4* was conjoined with exon 2 of *NUT* generating an in-frame fusion gene (Figure 2B) that skips exon 12 of *BRD4*, a short intervening gene *NOP10* in the opposite strand, and exon 1 of *NUT*. We also identified an alternative splicing event generating an independent, out-of-frame *BRD4*−*NUT* transcript using an intervening cryptic exon (supplementary Figure S4, available at *Annals of Oncology* online), but the pathogenic significance would be minimal. Similarly, in NMC2, we identified 12 genomic breakpoints involved in the *BRD3*−*NUT* rearrangement. Those breakpoints were located in chromosomes 3, 9 and 15 also generating a closed chain without local DNA copy number changes. Exon 11 of *BRD3* was truncated and connected with intron 4 of *NUT* (Figures 1B and 3A); this rearrangement generated fusion transcripts conjoining exon 10 of *BRD3* with exon 5 of *NUT* (Figure 3B). However, both ends of the rearranged DNA segment encompassing the *BRD3*−*NUT* were conjoined with a non-specific AATGG (centromeric) repeat, which made it difficult to reconstruct the complete pattern of the complex rearrangements using sequencing data only. Therefore, we further investigated the genome of the NMC2 cells with M-FISH (supplementary Figure S5, available at *Annals of Oncology* online) and BAC clones (BAC-FISH). Finally, we identified that the rearranged *NUT* gene was inserted into the repetitive region of the short arm of the acrocentric chromosome 15 (Figure 4). Of note, many rearrangements of submicroscopic segments (<10 Mb), including the DNA segment with the rearranged *NUT* gene in this case, were not apparent in conventional karyotyping. All the characteristics of these complex chromosomal rearrangements, i.e. the closed-chain pattern of rearrangement, clustered location of breakpoints, no evidence of segmental loss, no significant copy number change at the rearrangement junctions, and little sequence microhomology at the rearrangement breakpoints, indicate that the oncogenic *BRD3/4*−*NUT* rearrangements can be attributed to a single catastrophic event rather than a gradual accumulation, during the life history of the most recent common ancestor (MRCA) cell of the NMCs [15].

We wondered whether complex SVs rather than simple balanced reciprocal translocations underlay the *NUT* rearrangements even in the NMC cases with a simple t(15;19) karyotype. We sequenced the whole genome of Ty-82, one of the oldest and well-characterized NMC cell lines [13]. Interestingly, we found that the interchromosomal *BRD4*−*NUT* rearrangement was not simple but was combined with a local inversion and copy number amplification (from 2 to 3 copies) of a small (∼300 bp) intervening DNA segment (supplementary Figure S6, available at *Annals of Oncology* online). The local copy number change and sequence microhomology at the rearrangement junctions indicated that this complex rearrangement was likely generated by the microhomology-mediated break-induced replication mechanism, which has been frequently observed in complex genomic rearrangements [18].

### Somatic mutations and their mutational signatures

**Figure 5.**
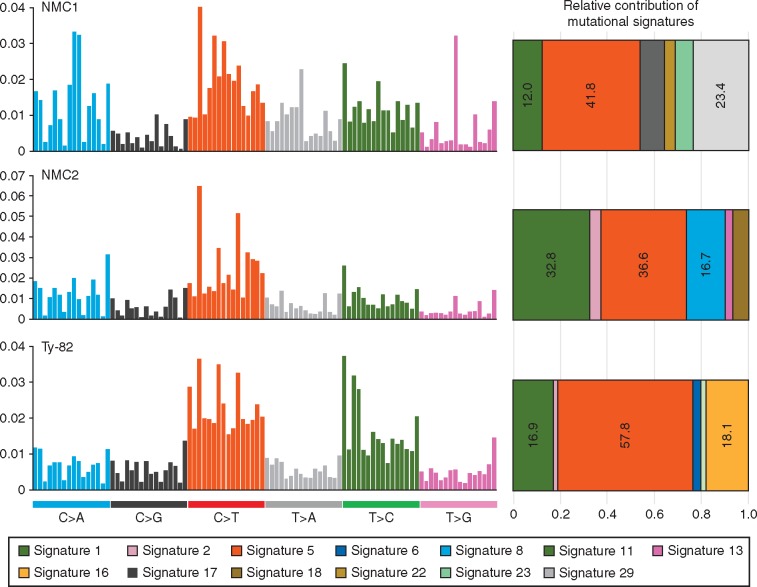
Mutational spectra of NMC cases and contributing signatures. The spectra of base substitutions in three NMC cases defined by the type of substitutions (i.e. C > A or C > T) and their adjacent bases contexts are plotted in histograms (left). The order of 96 trinucleotide contexts is according to the previous study by Alexandrov et al. [32]. From this data, mutational signatures contributing each case were extracted using the deconstructSigs package, and are depicted in bar graphs (right).

We next examined somatic point mutations including base substitutions and short indels. We identified 3368 and 3168 somatic base substitutions in NMC1 and NMC2, respectively (supplementary Table S4, available at *Annals of Oncology* online), with a mutation rate higher than 1.1/Mb. The crude mutation rate of NMC was higher than expected [19]; the burden of mutation in both NMC cases was comparable to other cancer types of different tissues, for example, breast cancers (median number of substitutions = 3492 from 560 whole-genome sequences) [20], low-grade gliomas and B-cell lymphomas [21]. To investigate the mutational processes that have been operative in the NMC cells, we analyzed the mutational signatures of NMC1, NMC2 and Ty-82 (Figure 5) [21]. A large portion of somatic base substitutions were attributable to signature 1 (dominantly C:G > T:A substitution at a CpG sequence context due to endogenous methyl-cytosine deamination) and signature 5 (dominantly T:A > C:G transition; unknown mechanism) in all three samples (53.8%, 69.4% and 74.7% of substitutions in NMC1, NMC2 and Ty-82, respectively). The proportion is comparable or even larger when compared with many other cancer types, i.e. 49.2% in breast cancers [20]. These two mutational signatures are known to have molecular clock-like properties, which may generate mutations throughout life at a more-or-less constant rate [22]. A recent study demonstrated that annual mutation rates of adult stem cells of colon, small intestine and liver were very similar: ∼40 point mutations (most of them were attributable to signatures 1 and 5) per year [23]. Overall, prevalent somatic mutations of those two molecular clock-like signatures in our NMCs (1811 for NMC1 and 2199 for NMC2) despite the short chronological ages at diagnosis (NMC1 and NMC2 were 34 and 33 years old, respectively, at diagnosis and Ty-82 was established from a 22-year-old female patient [13]) may represent either distinct physiology of NMC origin cells (i.e. higher number of cell division compared with other adult stem cells) or activation of clock-like mutational processes after malignant transformation.

Interestingly, we found a C:G > A:T substitution-dominant mutational signature (signature 29), which was known as tobacco chewing-related [21], in NMC1 (accounting for 23.4% of the somatic substitutions in the case). Because the smoking carcinogen induces a G > T transversion mutation only in the epithelial cells that have direct exposure to the carcinogen from tobacco [21], we conclude that the ancestral cells of NMC1 were located in tissues directly exposed to tobacco smoke at the pre-neoplastic stage. In the NMC2 genome, we found no evidence of tobacco-induced mutations, which was consistent with the never-smoking history of the patient. 16.7% of the substitutions were attributable to the unknown signature 8, and 7.3% were APOBEC-induced mutations (signatures 2 and 13) in NMC2. Ty-82 showed the highest percentage of signature 1 and 5 mutations (74.7%), which was consistent with the prolonged history of this cell line since 1992.

Of the >3000 somatic base substitutions in the NMC1 and NMC2 genomes, 18 and 24 changed the amino acids of protein-coding genes, respectively. However, no canonical oncogenes or tumor suppressor genes were affected. A total of 200 and 222 short indels were identified from NMC1 and NMC2, but only 3 from NMC1 (none of them were in the cancer-related genes), and none from NMC2 were located in the protein-coding sequences. These suggest that the functional contribution of somatic point mutations for neoplastic transformation is limited in the NMCs.

## Discussion

Chromoplexy is a unique pattern of complex genomic rearrangement first described in prostate cancers. Although the detailed molecular mechanism is currently unknown, it is notable that the chromoplexy frequently involves cancer driver genes in some cancer types, for example, ∼60% of prostate cancer cases harbor chromoplexy affecting one or more prostate cancer-related genes including *TMPRSS2*−*ERG* rearrangements [17]. In the present study, we found chromoplexy events generating the *bona fide* oncogene *BRD3/4*−*NUT* although not in all cases. It warrants further investigation whether previously described NMC cases with a non-canonical karyotype also involve chromoplexy in their *BRD3/4*−*NUT* rearrangements. We speculate that the involvement of a large number of DNA segments with highly expressed genes and remarkable absence of segmental loss may suggest the pathogenesis related with transcriptional process and involvement of recombinogenic enzymes as described for *TMPRSS2*−*ETS* rearrangements in a previous study [24]. Frequent involvement of cancer-related genes in chromoplexy may suggest this could be a common, and efficient way to generate driver oncogenic rearrangements, although they look highly complex.

It has been postulated that the origin cell of NMCs might be the primordial epithelial cells, most prevalent in the late teenage years in the midline anatomical structures [19]. If this is the case, our findings suggest that these primordial cells are likely to acquire many point mutations from endogenous cellular processes. However, the neoplastic transformation is mainly due to the pathognomonic *NUT* rearrangements obtained by a stochastic, single catastrophic process.

From the whole-genome and transcriptome sequencing of the NMC cases, this study provides insight on the pathogenesis of this devastating disease. Sequencing of a large cohort of NMCs may not be feasible because of the rarity of this tumor and also because of the unavailability of fresh tissues which are optimal for high-throughput sequencing. International collaborative efforts will be necessary for the comprehensive understanding of the pathogenesis and for the development of effective therapeutic modalities.

## Funding

This work was supported by Korea Health Technology R&D Project through the Korea Health Industry Development Institute (KHIDI), which was funded by the Ministry of Health & Welfare (HI14C1234; T.M.K., HI16C2387; Y.S.J.), a fellowship from the National Research Foundation of Korea (NRF-2013H1A2A1032691; J.K.L.), The Molecular Cytogenetics Core Facility at the Wellcome Trust Sanger Institute and Sanger investigators are funded by the Wellcome Trust (grant number WT098051; F.Y.). The dataset of this study is available at European Genome-phenome Archive (EGA; https://www.ebi.ac.uk/ega/home) with accession number EGAS00001001934.

## Disclosure

The authors have declared no conflicts of interest.

## Supplementary Material

Supplementary Data
