# Supplementary material for: Complex chromosomal rearrangements by single catastrophic pathogenesis in NUT midline carcinoma
Source: Ann Oncol. 2017 Feb 14;28(4):890–7. doi: 10.1093/annonc/mdw686 (PMC5378225; doi:10.1093/annonc/mdw686)
Supplement: Supplementary Data [file mdw686_supp.zip › mdw686-suppl_data/Supplementary Text.docx]

**SUPPLEMENTARY TEXT**

**MATERIALS AND METHODS**

**Whole-Genome Sequencing and Transcriptome Sequencing**

Genomic DNA and mRNA were extracted from the cell pellets of the early batch SNU-2972-1 and SNU-3178S cells and from Ty-82 cells (purchased from JCRB: #JCRB1330). For matched normal controls, genomic DNAs were obtained from the peripheral blood samples. Whole-genome sequencing libraries were generated according to the standard protocol of Illumina from 1 µg of genomic DNAs and subjected to high-throughput sequencing for 2 x 151 cycles on the HiSeq X machine (target coverage: 30X). An mRNA sequencing library was generated following the TruSeq protocol with 1 µg of mRNAs, and then sequenced for 2 x 100 cycles on the HiSeq 2500 machine (target throughput: 10 Gb).

**Variant Calling**

Reads were aligned to the reference human genome (GRCh37/hg19) by BWA MEM (v. 0.7.13), and the output sam files were converted into bam files and then sorted using Samtools (v. 1.3). PCR duplicates were marked with Picard and local realignment and base quality recalibration were performed using GATK (v. 3.5). Somatic base substitutions and small indels were investigated using Strelka (25) and MuTect (26), and base substitutions that were supported by both algorithms were used as consensus substitution sets. With Ty-82 for which a paired normal sequence was not available, we excluded all base substitutions that are listed in the 1000 genome, ExAC, or dbSNP database. Estimation of the tumor cell fraction and allele-specific copy number analysis were done with Sequenza (27). Large structural variations were identified using Delly2 (28). mRNA sequencing reads were mapped to the reference genome using TopHat2 (v. 2.1.1) and assembled using cufflinks (v. 2.2.1). Predicted sequences of *BRD3/4*−*NUT* in-frame transcripts are provided at the end of Supplementary Text.

**Supplementary References**

25. Saunders CT, Wong WS, Swamy S et al. Strelka: accurate somatic small-variant calling from sequenced tumor-normal sample pairs. Bioinformatics. 2012; 28: 1811-7.

26. Cibulskis K, Lawrence MS, Carter SL et al. Sensitive detection of somatic point mutations in impure and heterogeneous cancer samples. Nat Biotechnol. 2013; 31: 213-9.

27. Favero F, Joshi T, Marquard AM et al. Sequenza: allele-specific copy number and mutation profiles from tumor sequencing data. Ann Oncol. 2015; 26: 64-70.

28. Rausch T, Zichner T, Schlattl A et al. DELLY: structural variant discovery by integrated paired-end and split-read analysis. Bioinformatics. 2012; 28: i333-i9.

**LEGENDS FOR SUPPLEMENTARY FIGURES**

**Supplementary Figure 1. Conventional karyotyping of NMC cells**

Representative karyotypes of SNU-2972-1 (A) and SNU-3178S (B) are shown using conventional Giemsa staining. Red arrows indicate abnormal karyotypes.

**Supplementary Figure 2. M-FISH of NMC1**

The result of M-FISH in NMC1. Ten metaphase cells with clear image are shown in each column. Red boxes indicate abnormal karyotypes.

**Supplementary Figure 3. Cytogenetic validation of chromoplexy in NMC1**

Fluorescence *in situ* hybridization using the individual paint probes of chromosomes 15, 19 and 21 visualized the derivative chromosomes.

**Supplementary Figure 4. Alternative splicing of *BRD4*−*NUT* in NMC1**

Relative expression of *NUT* and *NOP10* are described. A novel alternative splicing event was observed in NMC1, in contrast to NMC2 and Ty-82. Cryptic splice sites in the downstream of *NOP10* are used to generate a 169 bp-sized intervening exon, resulting in frameshift transcription.

**Supplementary Figure 5. M-FISH of NMC2**

The result of M-FISH in NMC2. Ten metaphase cells with clear image are shown in each column. Red boxes indicate abnormal karyotypes.

**Supplementary Figure 6. *BRD4*−*NUT* rearrangement in Ty-82**

Rearrangement breakpoints of *BRD4*−*NUT* in Ty-82 cells and possible mechanism generating this rearrangement are described. Green and purple lines indicate progression of replication forks, and a dashed segment in green line indicates a template switch within chromosome 15. The size of microhomology bases at the breakpoints are also described.

**Predicted Sequences of *BRD3/4*−*NUT* In-Frame Fusion Transcripts**

**NMC1 (*BRD4*−*NUT*)**

attctttggaatactactgctagaagtctgacttaagacccagcttatgggccacatggcacccagctgcttctgcagagaaggcaggccactgatgggtacagcaaagtgtggtgctgctggccaagccaaagacccgtgtaggatgactgggcctctgccccttgtgggtgttgccactgtgcttgagtgcctggtgaagaatgtgatgggatcactagcatgtctgcggagagcggccctgggacgagattgagaaatctgccagtaatgggggatggactagaaacttcccaaatgtctacaacacaggcccaggcccaaccccagccagccaacgcagccagcaccaaccccccgcccccagagacctccaaccctaacaagcccaagaggcagaccaaccaactgcaatacctgctcagagtggtgctcaagacactatggaaacaccagtttgcatggcctttccagcagcctgtggatgccgtcaagctgaacctccctgattactataagatcattaaaacgcctatggatatgggaacaataaagaagcgcttggaaaacaactattactggaatgctcaggaatgtatccaggacttcaacactatgtttacaaattgttacatctacaacaagcctggagatgacatagtcttaatggcagaagctctggaaaagctcttcttgcaaaaaataaatgagctacccacagaagaaaccgagatcatgatagtccaggcaaaaggaagaggacgtgggaggaaagaaacagggacagcaaaacctggcgtttccacggtaccaaacacaactcaagcatcgactcctccgcagacccagacccctcagccgaatcctcctcctgtgcaggccacgcctcaccccttccctgccgtcaccccggacctcatcgtccagacccctgtcatgacagtggtgcctccccagccactgcagacgcccccgccagtgcccccccagccacaacccccacccgctccagctccccagcccgtacagagccacccacccatcatcgcggccaccccacagcctgtgaagacaaagaagggagtgaagaggaaagcagacaccaccacccccaccaccattgaccccattcacgagccaccctcgctgcccccggagcccaagaccaccaagctgggccagcggcgggagagcagccggcctgtgaaacctccaaagaaggacgtgcccgactctcagcagcacccagcaccagagaagagcagcaaggtctcggagcagctcaagtgctgcagcggcatcctcaaggagatgtttgccaagaagcacgccgcctacgcctggcccttctacaagcctgtggacgtggaggcactgggcctacacgactactgtgacatcatcaagcaccccatggacatgagcacaatcaagtctaaactggaggcccgtgagtaccgtgatgctcaggagtttggtgctgacgtccgattgatgttctccaactgctataagtacaaccctcctgaccatgaggtggtggccatggcccgcaagctccaggatgtgttcgaaatgcgctttgccaagatgccggacgagcctgaggagccagtggtggccgtgtcctccccggcagtgccccctcccaccaaggttgtggccccgccctcatccagcgacagcagcagcgatagctcctcggacagtgacagttcgactgatgactctgaggaggagcgagcccagcggctggctgagctccaggagcagctcaaagccgtgcacgagcagcttgcagccctctctcagccccagcagaacaaaccaaagaaaaaggagaaagacaagaaggaaaagaaaaaagaaaagcacaaaaggaaagaggaagtggaagagaataaaaaaagcaaagccaaggaacctcctcctaaaaagacgaagaaaaataatagcagcaacagcaatgtgagcaagaaggagccagcgcccatgaagagcaagccccctcccacgtatgagtcggaggaagaggacaagtgcaagcctatgtcctatgaggagaagcggcagctcagcttggacatcaacaagctccccggcgagaagctgggccgcgtggtgcacatcatccagtcacgggagccctccctgaagaattccaaccccgacgagattgaaatcgactttgagaccctgaagccgtccacactgcgtgagctggagcgctatgtcacctcctgtttgcggaagaaaaggaaacctcaagctgagaaagttgatgtgattgccggctcctccaagatgaagggcttctcgtcctcagagtcggagagctccagtgagtccagctcctctgacagcgaagactccgaaacagcatctgcattgccgggaccggatatgagcatgaaacctagtgccgccctgtctccatcccctgcacttccctttctcccaccaacttctgacccaccagaccacccacccagggagccacctccacagcccatcatgccttcagtattctctccagacaaccctctgatgctctctgctttccccagctcactgttggtgacaggggacgggggcccttgcctcagtggggctggggctggcaaggtcattgtcaaagtcaagacagaaggggggtcagctgagccctctcaaactcagaactttatccttactcagactgccctcaattcgactgccccgggcactccctgtggaggccttgagggtcctgcacctccatttgtgacagcatctaatgtgaagaccattctgccctctaaggctgttggtgtcagccaggagggtcctccaggccttccgcctcagcctccaccaccagttgctcaactggtccccattgtgcccctggaaaaagcttggccagggccacatgggacaaccggggaaggaggtcctgtggccactctatccaagccttccctaggtgaccgctccaaaatttccaaggacgtttatgagaacttccgtcagtggcagcgttacaaagccttggcccggaggcacctatcccagagtcctgacacagaagctctttcctgttttcttatcccagtgcttcgttccctggcccggctgaagcccactatgaccctggaggagggactgccattggctgtgcaggagtgggagcacaccagcaactttgaccggatgatcttttatgagatggcagaaaggttcatggagtttgaggctgaggagatgcagattcagaacacacagctgatgaatgggtctcagggcctgtctcctgcaacccctttgaaacttgatcctctagggcccctggcctctgaggtttgccagcagccagtgtacattccgaagaaggcagcctccaagacacgggccccccgccggcgtcagcgtaaagcccagagacctcctgctcctgaggcacccaaggagatcccaccagaagctgtgaaggagtatgttgacatcatggaatggctggtggggactcacttggccactggggagtcagatggaaaacaagaggaagaagggcagcagcaggaggaggaagggatgtatccagatccaggtctcctgagctacatcaatgagctgtgttctcagaaggtctttgtctccaaggtggaggctgtcattcaccctcaatttctggcagatctgctgtccccagaaaaacagagagatcccttggccttaattgaggagctagagcaagaagaaggactcactcttgcccagctggtccagaagcgactcatggccttggaagaggaggaagatgcagaggcgcctccaagtttcagtggcgctcagttggactcaagtccttctggttctgttgaggatgaagatggggatgggcggcttcggccctcacctgggcttcagggggctgggggcgccgcttgccttggaaaggtttcttcttcaggaaaacgggcaagagaagtgcatggtgggcaggagcaagccctagatagccccagagggatgcacagggatgggaacactctgccatcccccagcagctgggacctgcagccagaacttgcagctccacagggaactccgggacccttgggtgtggagaggagagggtctgggaaggttataaaccaggtatctctacatcaggatggccatctaggaggcgctgggcctcctgggcactgcctggtggctgataggacttcagaggctctgcccctttgttggcagggaggcttccagcctgagagcactcccagtttggatgctggacttgcagagctggctcctctgcaaggacaagggttagaaaagcaagtcctgggattgcagaaaggacaacaaacagggggtcgtggagtgcttcctcaagggaaggagcctttagcagtgccctgggaaggctcttcaggagccatgtggggagatgacagaggtacccccatggctcagagttatgatcagaatccttcccctagagcagctggggagagggacgatgtctgtctcagcccaggagtttggctgagcagtgagatggatgctgtaggcttggagctgcctgtacaaatagaggaggtcatagagagcttccaagttgagaagtgtgtaactgagtatcaggaaggctgccagggactgggctccaggggcaacatttccctgggtcctggagaaaccctagtacctggggatacggagagcagtgtgattccctgtggaggcacagttgcggcagctgccctagaaaagagaaactattgcagcttgccaggacctttgagggccaacagcccacccttgaggtccaaagaaaatcaagaacagagctgtgaaaccgtagggcatcccagtgatctgtgggcagaaggttgcttcccattgctagaaagtggtgattccacactggggtcttccaaagaaacccttccacccacatgccaaggcaatctccttatcatggggactgaggatgcctcctccttgcctgaagccagtcaagaggcagggagcagaggcaattccttttctcctctgttggaaaccatagaacctgtcaacatactagatgttaaagatgactgtggcctccaactaagggtcagcgaggacacctgcccactgaatgttcattcttatgacccccaaggagaaggcagggtggatcctgatctgtccaagcctaaaaaccttgctcctttacaagagagtcaggagtcttacacaactgggactcccaaagcaacatcttctcaccagggccttggaagcactttgcctagaaggggaaccaggaatgccatagttccgagagaaacttctgttagtaaaacacacaggtcagcagacagggccaaaggaaaggagaaaaagaaaaaggaagcagaggaagaggatgaggaactctccaactttgcttacctcttggcctctaaacttagcctctcaccaagggagcatcccctcagtcctcaccatgcctcaggaggtcagggcagccagagagcatcccacctgctccctgctggagcaaaaggccccagcaaacttccatatcctgttgccaagtctgggaagcgagctctagctggaggtccagcccctactgaaaagacaccccactcaggagctcaacttggggtccccagggagaaacccctagctctgggagtagttcgaccctcacagcctcgtaaaaggcggtgtgacagttttgtcacgggcagaaggaagaaacgacgtcgtagccagtagggagcagcgggaccatctgaccccacttgccagtccctaaaggtgggtgccccagagtagattccacccctgctgcccaccaatggagaatcccaatgttgaatctcatcccaatgttgttttgttgttctgcaaaagtggcaagcatggagagagaggtcagactggctaggctgcagggggaattacctttggaaggagctatatagaaaaaaaatgaataaagtgttttgttggaaa

**NMC2 (*BRD3*−*NUT*)**

tgccggggccggcgagccaaagaggagccggccgcgcgggccgggaggggacggccgccggagccgcgaggccaactgtcgcctggttgggcccggaaatgggacgtcgcgctttctcagggagcgtagaagcagccagggcctctccaagccgctgctgtgacagaaagtgagtgagctgccggaggatgtccaccgccacgacagtcgcccccgcggggatcccggcgaccccgggccctgtgaacccaccccccccggaggtctccaaccccagcaagcccggccgcaagaccaaccagctgcagtacatgcagaatgtggtggtgaagacgctctggaaacaccagttcgcctggcccttctaccagcccgtggacgcaatcaaattgaacctgccggattatcataaaataattaaaaacccaatggatatggggactattaagaagagactagaaaataattattattggagtgcaagcgaatgtatgcaggacttcaacaccatgtttacaaattgttacatttataacaagcccacagatgacatagtgctaatggcccaagctttagagaaaatttttctacaaaaagtggcccagatgccccaagaggaagttgaattattaccccctgctccaaagggcaaaggtcggaagccggctgcgggagcccagagcgcaggtacacagcaagtggcggccgtgtcctctgtctccccagcgaccccctttcagagcgtgccccccaccgtctcccagacgcccgtcatcgctgccacccctgtaccaaccatcactgcaaacgtcacgtcggtcccagtccccccagctgccgccccacctcctcctgccacacccatcgtccccgtggtccctcctacgccgcctgtcgtcaagaaaaagggcgtgaagcggaaagcagacacaaccactcccacgacgtcggccatcactgccagccggagtgagtcgcccccgccgttgtcagaccccaagcaggccaaagtggtggcccggcgggagagtggtggccgccccatcaagcctcccaagaaggacctggaggacggcgaggtgccccagcacgcaggcaagaagggcaagctgtcggagcacctacgctactgcgacagcatcctcagggagatgctatccaagaagcacgcggcctacgcctggcccttctacaagccagtggatgccgaggccctggagctgcacgactaccacgacatcatcaagcacccgatggacctcagcaccgtgaaaaggaagatggatggccgagagtacccagacgcacagggctttgctgctgatgtccggctgatgttctcgaattgctacaaatacaatcccccagaccacgaggttgtggccatggcccggaagctccaggacgtgtttgagatgaggtttgccaagatgccagatgagcccgtggaggcaccggcgctgcctgcccccgcggcccccatggtgagcaagggcgctgagagcagccgtagcagtgaggagagctcttcggactcaggcagctcggactcggaggaggagcgggccaccaggctggcggagctgcaggagcagctgaaggccgtgcacgagcagctggccgccctgtctcaggccccagtaaacaaaccaaagaagaagaaggagaagaaggagaaggagaagaagaagaaggacaaggagaaggagaaggagaagcacaaagtgaaggccgaggaagagaagaaggccaaggtggctccgcctgccaagcaggctcagcagaagaaggctcctgccaagaaggccaacagcacgaccacggccggcagacagctgaagaaaggcggcaagcaggcatctgcctcctacgactcagaggaagaggaggagggcctgcccatgagctacgatgaaaagcgccagcttagcctggacatcaaccggctgcccggggagaagctgggccgggtagtgcacatcatccaatctcgggagccctcgctcagggactccaaccccgacgagatagaaattgactttgagactctgaaacccaccactttgcgggaactggagagatatgtcaagtcttgtttacagaaaaagcaaaggaaaccgttcttgtacattccgaagaaggcagcctccaagacacgggccccccgccggcgtcagcgtaaagcccagagacctcctgctcctgaggcacccaaggagatcccaccagaagctgtgaaggagtatgttgacatcatggaatggctggtggggactcacttggccactggggagtcagatggaaaacaagaggaagaagggcagcagcaggaggaggaagggatgtatccagatccaggtctcctgagctacatcaatgagctgtgttctcagaaggtctttgtctccaaggtggaggctgtcattcaccctcaatttctggcagatctgctgtccccagaaaaacagagagatcccttggccttaattgaggagctagagcaagaagaaggactcactcttgcccagctggtccagaagcgactcatggccttggaagaggaggaagatgcagaggcgcctccaagtttcagtggcgctcagttggactcaagtccttctggttctgttgaggatgaagatggggatgggcggcttcggccctcacctgggcttcagggggctgggggcgccgcttgccttggaaaggtttcttcttcaggaaaacgggcaagagaagtgcatggtgggcaggagcaagccctagatagccccagagggatgcacagggatgggaacactctgccatcccccagcagctgggacctgcagccagaacttgcagctccacagggaactccgggacccttgggtgtggagaggagagggtctgggaaggttataaaccaggtatctctacatcaggatggccatctaggaggcgctgggcctcctgggcactgcctggtggctgataggacttcagaggctctgcccctttgttggcagggaggcttccagcctgagagcactcccagtttggatgctggacttgcagagctggctcctctgcaaggacaagggttagaaaagcaagtcctgggattgcagaaaggacaacaaacagggggtcgtggagtgcttcctcaagggaaggagcctttagcagtgccctgggaaggctcttcaggagccatgtggggagatgacagaggtacccccatggctcagagttatgatcagaatccttcccctagagcagctggggagagggacgatgtctgtctcagcccaggagtttggctgagcagtgagatggatgctgtaggcttggagctgcctgtacaaatagaggaggtcatagagagcttccaagttgagaagtgtgtaactgagtatcaggaaggctgccagggactgggctccaggggcaacatttccctgggtcctggagaaaccctagtacctggggatacggagagcagtgtgattccctgtggaggcacagttgcggcagctgccctagaaaagagaaactattgcagcttgccaggacctttgagggccaacagcccacccttgaggtccaaagaaaatcaagaacagagctgtgaaaccgtagggcatcccagtgatctgtgggcagaaggttgcttcccattgctagaaagtggtgattccacactggggtcttccaaagaaacccttccacccacatgccaaggcaatctccttatcatggggactgaggatgcctcctccttgcctgaagccagtcaagaggcagggagcagaggcaattccttttctcctctgttggaaaccatagaacctgtcaacatactagatgttaaagatgactgtggcctccaactaagggtcagcgaggacacctgcccactgaatgttcattcttatgacccccaaggagaaggcagggtggatcctgatctgtccaagcctaaaaaccttgctcctttacaagagagtcaggagtcttacacaactgggactcccaaagcaacatcttctcaccagggccttggaagcactttgcctagaaggggaaccaggaatgccatagttccgagagaaacttctgttagtaaaacacacaggtcagcagacagggccaaaggaaaggagaaaaagaaaaaggaagcagaggaagaggatgaggaactctccaactttgcttacctcttggcctctaaacttagcctctcaccaagggagcatcccctcagtcctcaccatgcctcaggaggtcagggcagccagagagcatcccacctgctccctgctggagcaaaaggccccagcaaacttccatatcctgttgccaagtctgggaagcgagctctagctggaggtccagcccctactgaaaagacaccccactcaggagctcaacttggggtccccagggagaaacccctagctctgggagtagttcgaccctcacagcctcgtaaaaggcggtgtgacagttttgtcacgggcagaaggaagaaacgacgtcgtagccagtagggagcagcgggaccatctgaccccacttgccagtccctaaaggtgggtgccccagagtagattccacccctgctgcccaccaatggagaatcccaatgttgaatctcatcccaatgttgttttgttgttctgcaaaagtggcaagcatggagagagaggtcagactggctaggctgcagggggaattacctttggaaggagctatatagaaaaaaaatgaataaagtgttttgttggaaaat

**Ty-82 (*BRD4*−*NUT*)**

attctttggaatactactgctagaagtctgacttaagacccagcttatgggccacatggcacccagctgcttctgcagagaaggcaggccactgatgggtacagcaaagtgtggtgctgctggccaagccaaagacccgtgtaggatgactgggcctctgccccttgtgggtgttgccactgtgcttgagtgcctggtgaagaatgtgatgggatcactagcatgtctgcggagagcggccctgggacgagattgagaaatctgccagtaatgggggatggactagaaacttcccaaatgtctacaacacaggcccaggcccaaccccagccagccaacgcagccagcaccaaccccccgcccccagagacctccaaccctaacaagcccaagaggcagaccaaccaactgcaatacctgctcagagtggtgctcaagacactatggaaacaccagtttgcatggcctttccagcagcctgtggatgccgtcaagctgaacctccctgattactataagatcattaaaacgcctatggatatgggaacaataaagaagcgcttggaaaacaactattactggaatgctcaggaatgtatccaggacttcaacactatgtttacaaattgttacatctacaacaagcctggagatgacatagtcttaatggcagaagctctggaaaagctcttcttgcaaaaaataaatgagctacccacagaagaaaccgagatcatgatagtccaggcaaaaggaagaggacgtgggaggaaagaaacagggacagcaaaacctggcgtttccacggtaccaaacacaactcaagcatcgactcctccgcagacccagacccctcagccgaatcctcctcctgtgcaggccacgcctcaccccttccctgccgtcaccccggacctcatcgtccagacccctgtcatgacagtggtgcctccccagccactgcagacgcccccgccagtgcccccccagccacaacccccacccgctccagctccccagcccgtacagagccacccacccatcatcgcggccaccccacagcctgtgaagacaaagaagggagtgaagaggaaagcagacaccaccacccccaccaccattgaccccattcacgagccaccctcgctgcccccggagcccaagaccaccaagctgggccagcggcgggagagcagccggcctgtgaaacctccaaagaaggacgtgcccgactctcagcagcacccagcaccagagaagagcagcaaggtctcggagcagctcaagtgctgcagcggcatcctcaaggagatgtttgccaagaagcacgccgcctacgcctggcccttctacaagcctgtggacgtggaggcactgggcctacacgactactgtgacatcatcaagcaccccatggacatgagcacaatcaagtctaaactggaggcccgtgagtaccgtgatgctcaggagtttggtgctgacgtccgattgatgttctccaactgctataagtacaaccctcctgaccatgaggtggtggccatggcccgcaagctccaggatgtgttcgaaatgcgctttgccaagatgccggacgagcctgaggagccagtggtggccgtgtcctccccggcagtgccccctcccaccaaggttgtggccccgccctcatccagcgacagcagcagcgatagctcctcggacagtgacagttcgactgatgactctgaggaggagcgagcccagcggctggctgagctccaggagcagctcaaagccgtgcacgagcagcttgcagccctctctcagccccagcagaacaaaccaaagaaaaaggagaaagacaagaaggaaaagaaaaaagaaaagcacaaaaggaaagaggaagtggaagagaataaaaaaagcaaagccaaggaacctcctcctaaaaagacgaagaaaaataatagcagcaacagcaatgtgagcaagaaggagccagcgcccatgaagagcaagccccctcccacgtatgagtcggaggaagaggacaagtgcaagcctatgtcctatgaggagaagcggcagctcagcttggacatcaacaagctccccggcgagaagctgggccgcgtggtgcacatcatccagtcacgggagccctccctgaagaattccaaccccgacgagattgaaatcgactttgagaccctgaagccgtccacactgcgtgagctggagcgctatgtcacctcctgtttgcggaagaaaaggaaacctcaagctgagaaagttgatgtgattgccggctcctccaagatgaagggcttctcgtcctcagagtcggagagctccagtgagtccagctcctctgacagcgaagactccgaaacagagatggctccgaagtcaaaaaagaaggggcaccccgggagggagcagaagaagcaccatcatcaccaccatcagcagatgcagcaggccccggctcctgtgccccagcagccgcccccgcctccccagcagcccccaccgcctccacctccgcagcagcaacagcagccgccacccccgcctcccccaccctccatgccgcagcaggcagccccggcgatgaagtcctcgcccccacccttcattgccacccaggtgcccgtcctggagccccagctcccaggcagcgtctttgaccccatcggccacttcacccagcccatcctgcacctgccgcagcctgagctgccccctcacctgccccagccgcctgagcacagcactccaccccatctcaaccagcacgcagtggtctctcctccagcatctgcattgccgggaccggatatgagcatgaaacctagtgccgccctgtctccatcccctgcacttccctttctcccaccaacttctgacccaccagaccacccacccagggagccacctccacagcccatcatgccttcagtattctctccagacaaccctctgatgctctctgctttccccagctcactgttggtgacaggggacgggggcccttgcctcagtggggctggggctggcaaggtcattgtcaaagtcaagacagaaggggggtcagctgagccctctcaaactcagaactttatccttactcagactgccctcaattcgactgccccgggcactccctgtggaggccttgagggtcctgcacctccatttgtgacagcatctaatgtgaagaccattctgccctctaaggctgttggtgtcagccaggagggtcctccaggccttccgcctcagcctccaccaccagttgctcaactggtccccattgtgcccctggaaaaagcttggccagggccacatgggacaaccggggaaggaggtcctgtggccactctatccaagccttccctaggtgaccgctccaaaatttccaaggacgtttatgagaacttccgtcagtggcagcgttacaaagccttggcccggaggcacctatcccagagtcctgacacagaagctctttcctgttttcttatcccagtgcttcgttccctggcccggctgaagcccactatgaccctggaggagggactgccattggctgtgcaggagtgggagcacaccagcaactttgaccggatgatcttttatgagatggcagaaaggttcatggagtttgaggctgaggagatgcagattcagaacacacagctgatgaatgggtctcagggcctgtctcctgcaacccctttgaaacttgatcctctagggcccctggcctctgaggtttgccagcagccagtgtacattccgaagaaggcagcctccaagacacgggccccccgccggcgtcagcgtaaagcccagagacctcctgctcctgaggcacccaaggagatcccaccagaagctgtgaaggagtatgttgacatcatggaatggctggtggggactcacttggccactggggagtcagatggaaaacaagaggaagaagggcagcagcaggaggaggaagggatgtatccagatccaggtctcctgagctacatcaatgagctgtgttctcagaaggtctttgtctccaaggtggaggctgtcattcaccctcaatttctggcagatctgctgtccccagaaaaacagagagatcccttggccttaattgaggagctagagcaagaagaaggactcactcttgcccagctggtccagaagcgactcatggccttggaagaggaggaagatgcagaggcgcctccaagtttcagtggcgctcagttggactcaagtccttctggttctgttgaggatgaagatggggatgggcggcttcggccctcacctgggcttcagggggctgggggcgccgcttgccttggaaaggtttcttcttcaggaaaacgggcaagagaagtgcatggtgggcaggagcaagccctagatagccccagagggatgcacagggatgggaacactctgccatcccccagcagctgggacctgcagccagaacttgcagctccacagggaactccgggacccttgggtgtggagaggagagggtctgggaaggttataaaccaggtatctctacatcaggatggccatctaggaggcgctgggcctcctgggcactgcctggtggctgataggacttcagaggctctgcccctttgttggcagggaggcttccagcctgagagcactcccagtttggatgctggacttgcagagctggctcctctgcaaggacaagggttagaaaagcaagtcctgggattgcagaaaggacaacaaacagggggtcgtggagtgcttcctcaagggaaggagcctttagcagtgccctgggaaggctcttcaggagccatgtggggagatgacagaggtacccccatggctcagagttatgatcagaatccttcccctagagcagctggggagagggacgatgtctgtctcagcccaggagtttggctgagcagtgagatggatgctgtaggcttggagctgcctgtacaaatagaggaggtcatagagagcttccaagttgagaagtgtgtaactgagtatcaggaaggctgccagggactgggctccaggggcaacatttccctgggtcctggagaaaccctagtacctggggatacggagagcagtgtgattccctgtggaggcacagttgcggcagctgccctagaaaagagaaactattgcagcttgccaggacctttgagggccaacagcccacccttgaggtccaaagaaaatcaagaacagagctgtgaaaccgtagggcatcccagtgatctgtgggcagaaggttgcttcccattgctagaaagtggtgattccacactggggtcttccaaagaaacccttccacccacatgccaaggcaatctccttatcatggggactgaggatgcctcctccttgcctgaagccagtcaagaggcagggagcagaggcaattccttttctcctctgttggaaaccatagaacctgtcaacatactagatgttaaagatgactgtggcctccaactaagggtcagcgaggacacctgcccactgaatgttcattcttatgacccccaaggagaaggcagggtggatcctgatctgtccaagcctaaaaaccttgctcctttacaagagagtcaggagtcttacacaactgggactcccaaagcaacatcttctcaccagggccttggaagcactttgcctagaaggggaaccaggaatgccatagttccgagagaaacttctgttagtaaaacacacaggtcagcagacagggccaaaggaaaggagaaaaagaaaaaggaagcagaggaagaggatgaggaactctccaactttgcttacctcttggcctctaaacttagcctctcaccaagggagcatcccctcagtcctcaccatgcctcaggaggtcagggcagccagagagcatcccacctgctccctgctggagcaaaaggccccagcaaacttccatatcctgttgccaagtctgggaagcgagctctagctggaggtccagcccctactgaaaagacaccccactcaggagctcaacttggggtccccagggagaaacccctagctctgggagtagttcgaccctcacagcctcgtaaaaggcggtgtgacagttttgtcacgggcagaaggaagaaacgacgtcgtagccagtagggagcagcgggaccatctgaccccacttgccagtccctaaaggtgggtgccccagagtagattccacccctgctgcccaccaatggagaatcccaatgttgaatctcatcccaatgttgttttgttgttctgcaaaagtggcaagcatggagagagaggtcagactggctaggctgcagggggaattacctttggaaggagctatatagaaaaaaaatgaataaagtgttttgttggaaaatgctc
