# Supplementary figures and images for: Complex chromosomal rearrangements by single catastrophic pathogenesis in NUT midline carcinoma
Source: Ann Oncol. 2017 Feb 14;28(4):890–7. doi: 10.1093/annonc/mdw686 (PMC5378225; doi:10.1093/annonc/mdw686)

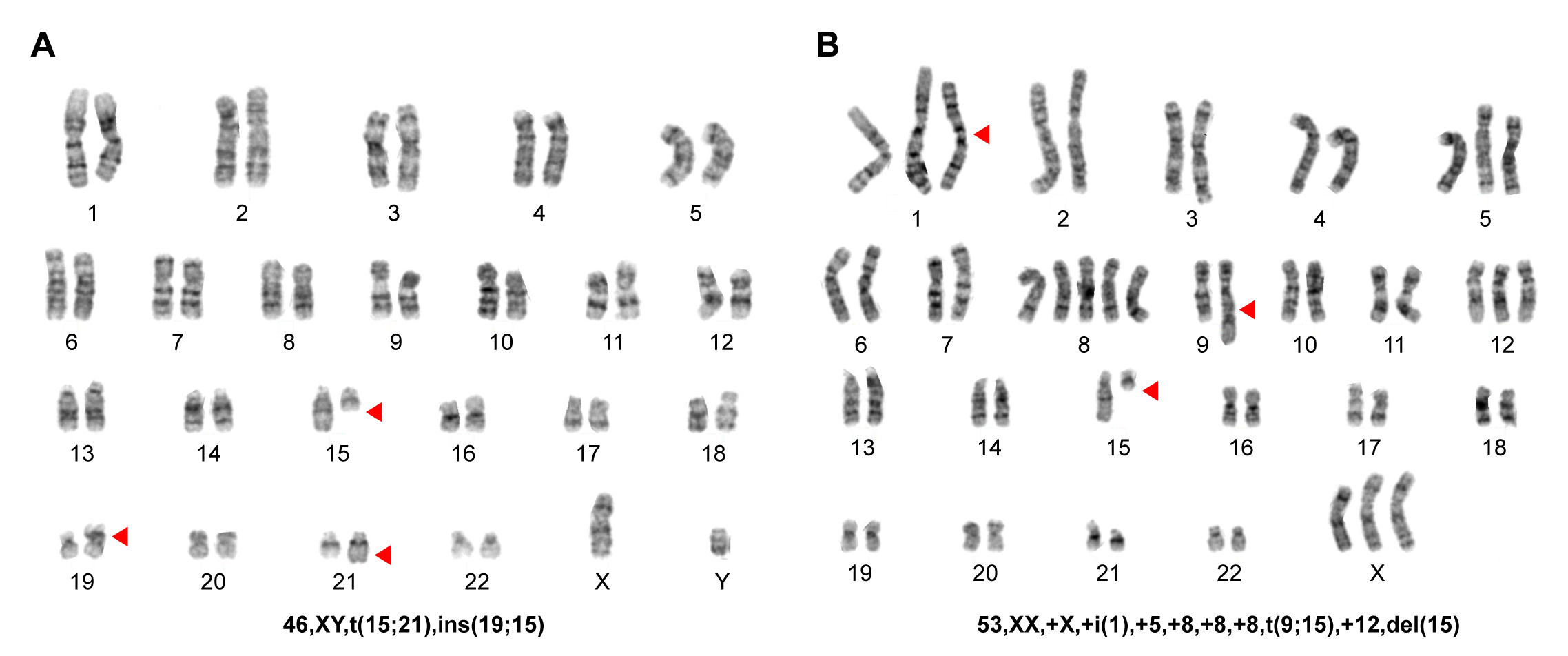

Supplement: Supplementary Data [file mdw686_supp.zip › mdw686-suppl_data/Supplementary_Figure1.tif]

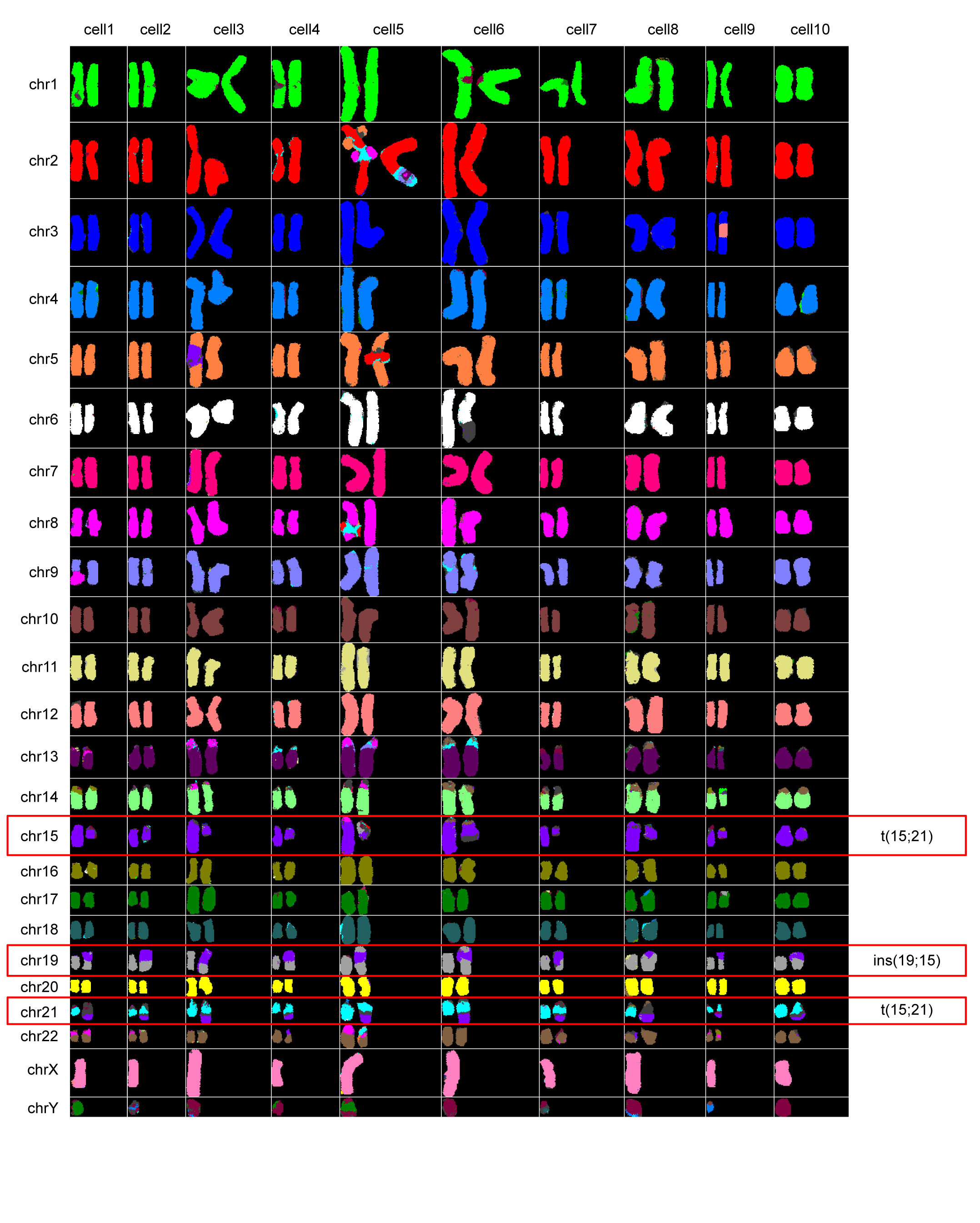

Supplement: Supplementary Data [file mdw686_supp.zip › mdw686-suppl_data/Supplementary_Figure2.tif]

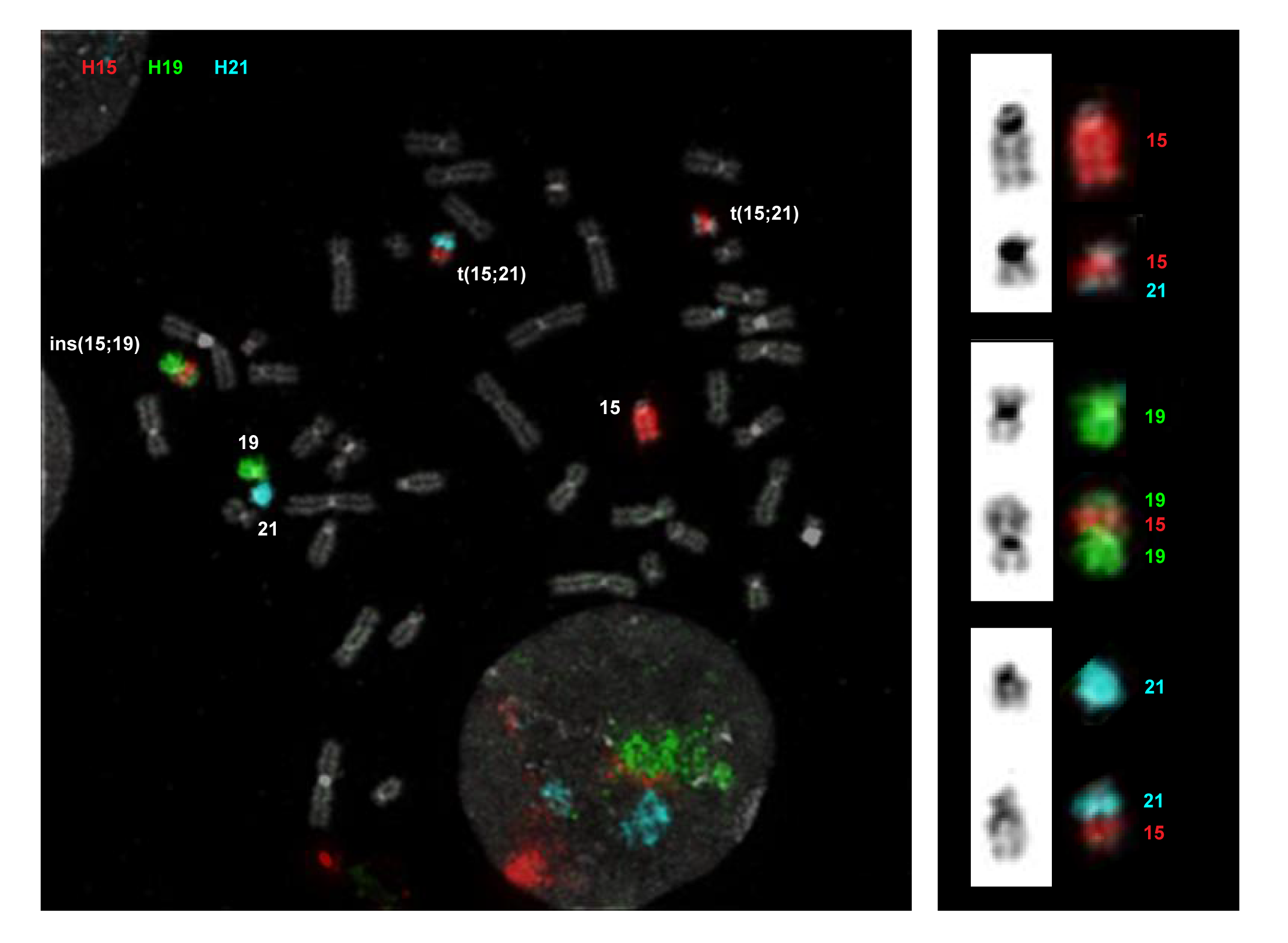

Supplement: Supplementary Data [file mdw686_supp.zip › mdw686-suppl_data/Supplementary_Figure3.tif]

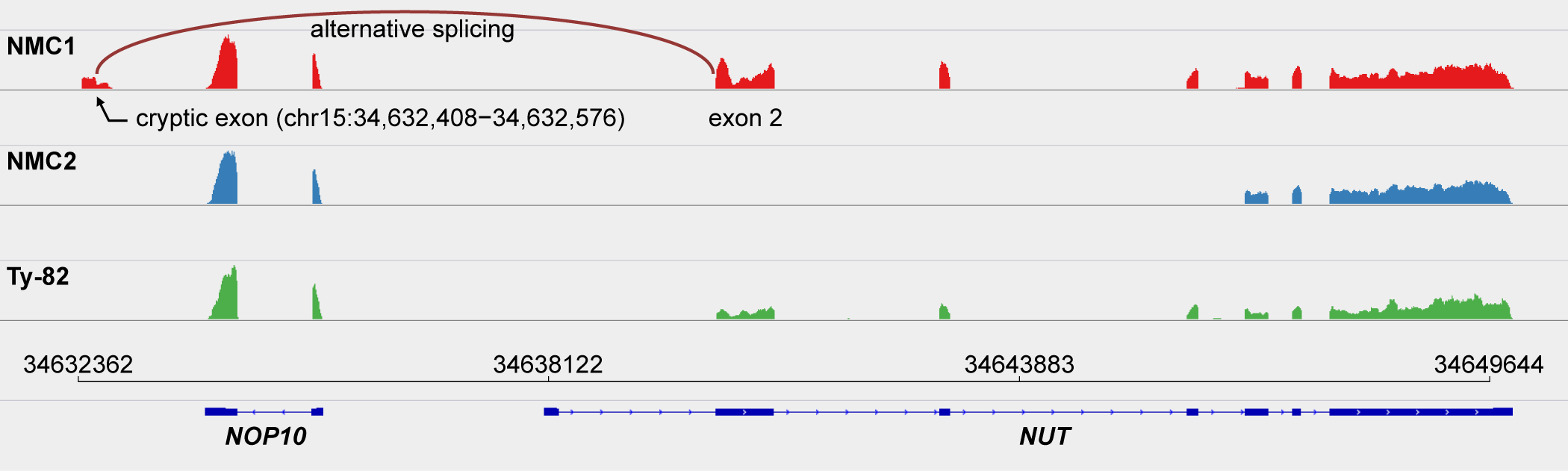

Supplement: Supplementary Data [file mdw686_supp.zip › mdw686-suppl_data/Supplementary_Figure4.tif]

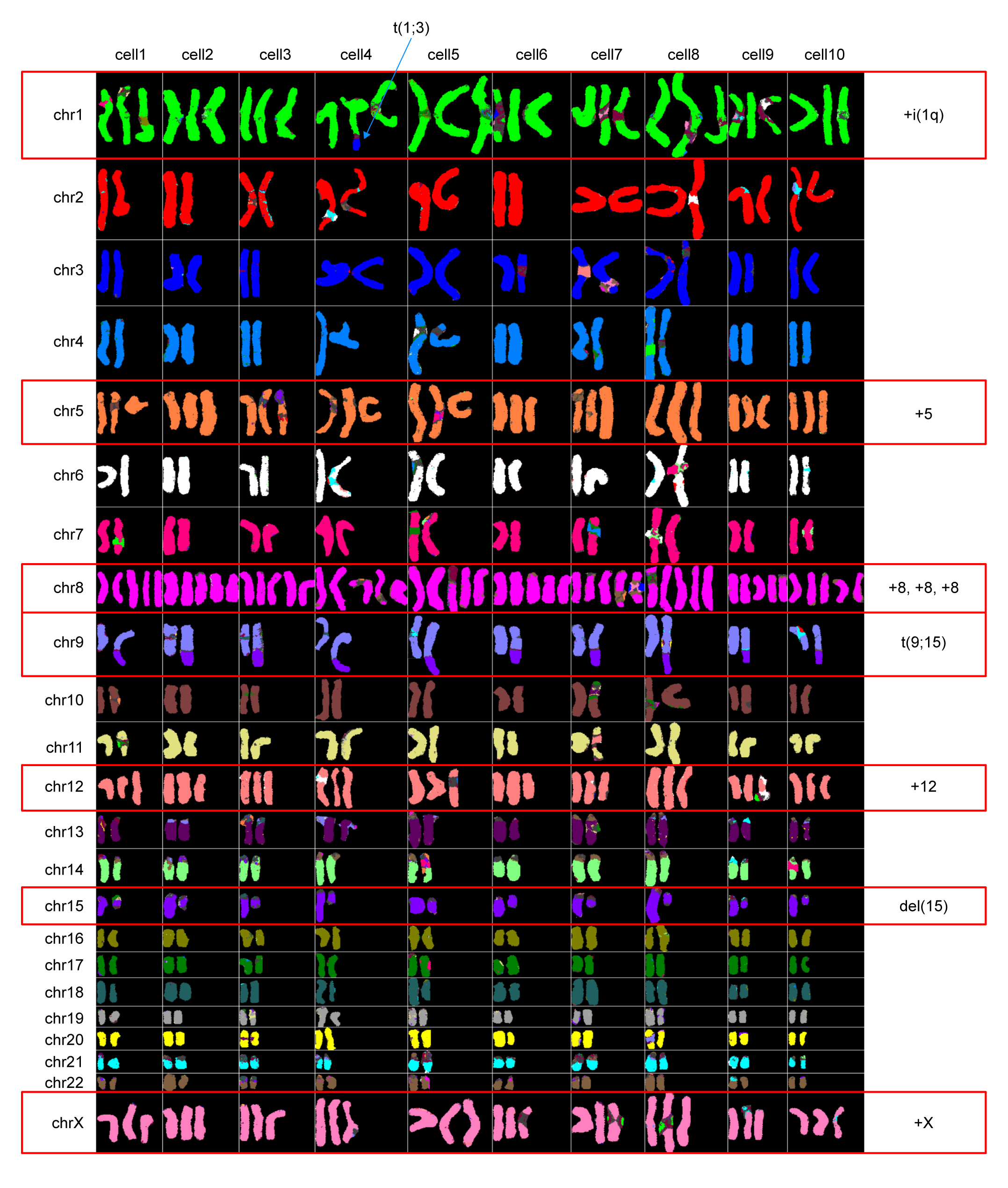

Supplement: Supplementary Data [file mdw686_supp.zip › mdw686-suppl_data/Supplementary_Figure5.tif]

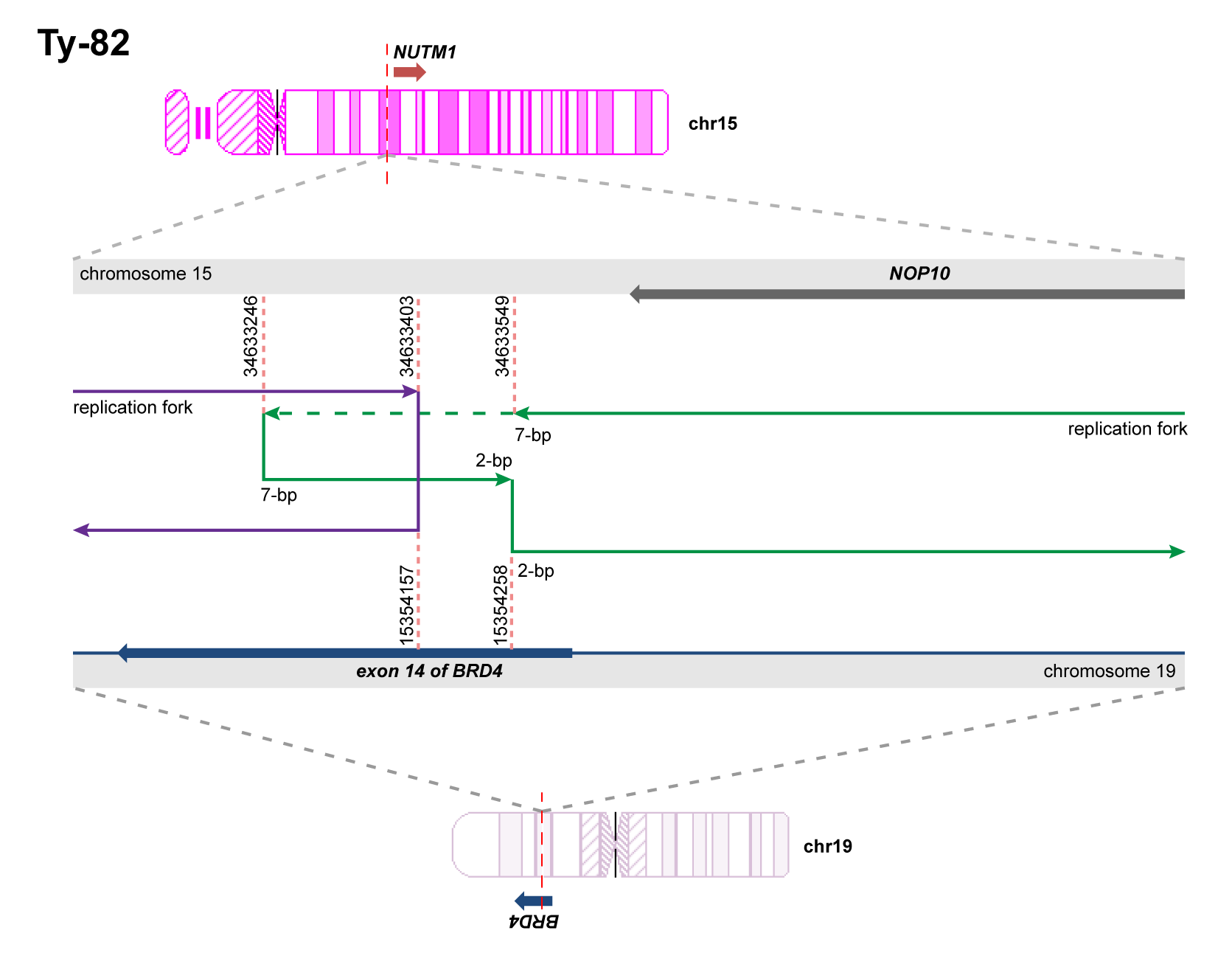

Supplement: Supplementary Data [file mdw686_supp.zip › mdw686-suppl_data/Supplementary_Figure6.tif]
